# The quality of denominator data in surgical site infection surveillance versus administrative data in Norway 2005–2010

**DOI:** 10.1186/s12879-015-1289-x

**Published:** 2015-11-30

**Authors:** Hege Line Løwer, Hanne-Merete Eriksen, Preben Aavitsland, Finn Egil Skjeldestad

**Affiliations:** Norwegian Institute of Public Health, Department of Infectious Disease Epidemiology, Oslo, Norway; Epidemi, Lasarettet, Kristiansand, Norway; Faculty of Health Sciences, Department of Community Medicine, Research Group Epidemiology of Chronic Diseases, UiT The Arctic University of Norway, Tromsø, Norway

**Keywords:** Electronic surveillance, Register data, Incidence, Infection control, Completeness, Representativeness

## Abstract

**Background:**

High quality of surveillance systems for surgical site infections (SSIs) is the key to their usefulness. The Norwegian Surveillance System for Antibiotic Consumption and Healthcare-Associated Infections (NOIS) was introduced by regulation in 2005, and is based largely on automated extraction of data from underlying systems in the hospitals.

**Methods:**

This study investigates the quality of NOIS-SSI’s denominator data by evaluating completeness, representativeness and accuracy compared with de-identified administrative data for 2005–2010. Comparisons were made by region, hospital type and size, age and sex for 4 surgical procedures.

**Results:**

The completeness of NOIS improved from 29.2 % in 2005 to 79.8 % in 2010. NOIS-SSI became representative over time for most procedures by hospital size and type, but not by region. It was representative by age and sex for all years and procedures. Accuracy was good for all years and procedures by all explanatory variables.

**Conclusions:**

A flexible and incremental implementation strategy has encouraged the development of computer-based surveillance systems in the hospitals which gives good accuracy, but the same strategy has adversely affected the completeness and representativeness of the denominator data. For the purpose of evaluating risk factors and implementing prevention and precautionary measures in the individual hospitals, representativeness seems sufficient, but for benchmarking and/or public reporting it is not good enough.

## Background

Surveillance of surgical site infections (SSIs) is increasingly regarded as a cornerstone in infection prevention. Many hospitals and countries have successfully implemented surveillance systems [1]. High quality of the systems is a prerequisite for their usefulness. National surveillance of SSIs in Norway was established with the Norwegian Surveillance System for Antibiotic Consumption and Healthcare-Associated Infections (NOIS) Act [2] in 2005, and we have earlier reported in detail on the rationale and functioning of this system [3, 4]. NOIS is based on the Hospitals in Europe Link for Infection Control through Surveillance [5] which was transferred to the European Centre for Disease Prevention and Control (ECDC) [6], and the definitions from the Centers for Disease Control and Prevention’s National Healthcare Safety Network [7].

Describing and evaluating the performance of a surveillance system is key to understanding its potential usefulness for public health authorities, hospitals, surgeons and hospital epidemiologists [8]. Validating the quality of the denominator data is important in order to ensure correct incidence rates and proportions. The objective of this study is to investigate denominator data quality by comparing surgical site infection surveillance data from NOIS-SSI with administrative data from the Norwegian Patient Register (NPR). We compare de-identified denominator data for the years 2005–2010 on an aggregated level in order to identify possible discrepancies in terms of completeness, representativeness and accuracy, and to recommend improvements.

## Methods

NPR was established in 1997 and contains information on all patients who receive specialist health care in Norway. Upon treatment in a hospital, an outpatient clinic or by a contracted private specialist, a series of data are recorded at the treatment site and transmitted to NPR three times a year. The objective of NPR is to form a basis for administration, management and quality assurance in specialist health care services, including financing and funding hospitals [9]. It is considered to be the complete database for hospital care in Norway [10]. NPR-data are harvested electronically from the hospital electronic health records (EHR). It is operated by the Norwegian Directorate of Health. The NPR-data relevant to the present study include variables for all admissions related to the procedure under observation: Patient identifier (de-identified), procedure code (Nordic Medico-Statistical Committee’s Classification of Surgical Procedures (NCSP)) [11], dates and times of admission, discharge and procedure, year of birth, sex, and hospital identifier.

NOIS was established in 2005 and is a national, mandatory surveillance system for health-care institutions [2]. The objective of the system is to describe the occurrence of healthcare-associated infections by time and other characteristics, detect outbreaks, provide a basis for preventive measures, and to evaluate such measures. It is coordinated by the Norwegian Institute of Public Health (NIPH) in collaboration with the hospitals. The first NOIS-module encompasses SSIs following several common surgical procedures, and is described in-depth in our previous publication [3]. Data are collected during an annual 3-month surveillance period (September-November). The data are de-identified by replacing the personal identifier with a serial number before the annual submission to the NIPH. The surveillance system relies to a great extent on automatic extraction of patient data from EHRs. There are three major suppliers of electronic infection control modules (ICMs) in use in Norway. In addition some hospitals have self-developed systems, some have manual systems and some have a combined manual and electronic system.

The following NCSP surgical procedures are included in this study (in order of priority in NOIS-SSI): coronary artery bypass graft (CABG), cesarean section (CSEC), hip arthroplasty (HPRO) and cholecystectomy (CHOL). During the first few years of NOIS-SSI, exemption from submitting surveillance data was given to hospitals so that they could establish suitable ICMs. Through 2009 hospitals were required to submit data from at least one of the surgical procedures under surveillance, and from 2010 and onwards at least two procedures [3]. Mixed CABG procedures (where aorta or ventricle surgery where performed in addition to bypass) were excluded in 2008 and mixed CHOL procedures (where other procedures are performed during the same surgery) were excluded in 2007 and 2008. NOIS-SSI includes data on the following variables of interest for this study; dates of admission, discharge and surgery, NCSP codes, age, sex, and hospital identifier.

We define a hospital as a single physical unit/location. A health care trust is a legal entity, often including several hospitals. There is a trend towards hospitals reporting data on a trust level. This causes the “hospital type” to be an ambiguous categorization over time, as one trust may include several different hospital types in the latter years. We have manually categorized hospitals according to ECDC classifications [6] as follows: primary (district hospital), secondary (provincial hospital), tertiary (university hospital), and specialized (non-profit/idealistic, private, contracted hospitals that mostly perform elective surgery within certain procedure types single specialty). Hospital size was also manually categorized and is influenced by the same issues as hospital type with regard to reporting on a trust level the latter years. Regions are designated according to the official categories, South-East, West, Central and North. Type of ICM was manually coded into four categories according to whether the NOIS-SSI data for a specific year was generated from of one of the three ICM suppliers (anonymized as A, B or C to protect the identity of individual hospitals), or from a manual or in-house system (other).

NOIS-SSI contains the patient’s actual age in years on the date of surgery, but the NPR-data only provides the year of birth. To correct for this, we calculated age by generating pseudo-random birth months (1–12) and days (1–28) for the NPR procedures in order to spread the patients evenly throughout the year.

In surveillance of SSIs, the denominator is the number of surgical procedures performed. One patient may undergo several procedures, such bilateral or staged hip replacement which counts as 2 procedures. The NPR-data received had one record per admission related to the surgical procedure. We converted these to one record per procedure based on the patient identifier, year of birth, sex, hospital identifier and date of surgery in order to make them comparable to SSI surveillance data. We were unable to account for bilateral hip replacements using this method, but such procedures were quite rare in Norway (0.22 % of total hip arthroplasties in 2005-2010 [12]) and would affect our outcomes minimally. Missing procedure dates in NPR (especially 2009) were substituted by date of admission. We excluded procedures which were duplicates, had invalid surgical procedure codes or were from private clinics with inconsistent data in both registers. In addition we excluded procedures from NOIS-SSI which were outside the 3-month surveillance window, and procedures from NPR from outside 2005–2010. NOIS-SSI data were appended to NPR data for data analysis purposes.

We evaluated the data quality of NOIS-SSI with regard to the completeness, representativeness and accuracy of the denominator data compared with NPR. We defined completeness as the total number of procedures in NOIS-SSI divided by the total number of procedures in NPR during the 3-month surveillance period for each procedure and year. Representativeness was assessed by comparing the distribution of data in NOIS-SSI with the distribution of data in NPR by hospital type and size, region, age and sex for each procedure and year. We defined accuracy as the agreement of data from hospitals and months which were present in both registers. We thus excluded data from hospitals or months which were not present in both registers from the comparison and divided the number of procedures in NOIS-SSI by NPR. We further compared the distributions in the two registers by the same variables as for representativeness. In addition we evaluated the accuracy based on the type of ICM used for collecting NOIS-SSI data. Frequencies were calculated for each of the surgical categories for each year, the whole period, and for each included variable. NOIS-SSI was evaluated against NPR in terms of percentages and chi-squared analysis. All data cleaning and analysis was done using Stata v.13 (Stata Statistical Software, College Station, TX). Access to de-identified data was granted to us at the discretion of the data proprietor in accordance with both registers’ acts. The study has been approved by the South East Regional Committee for Medical and Health Research Ethics, and the Norwegian Data Protection Authority has been notified. Patient consent is not required, as both NPR and NOIS are national health registers governed by separate acts.

## Results

After data cleaning 162,509 procedures remained from NPR for 2005–2010, whereof 45,347 (27.9 %) from September - November. From NOIS-SSI, 26,250 procedures were included from September-November of 2005–2010.

Table 1 shows completeness as the number of procedures submitted to NOIS-SSI divided by the total number of procedures in NPR for the 3-month surveillance period in 2005–2010. For the whole period, NOIS-SSI encompassed 57.9 % of the total number of surgical procedures in NPR. The overall completeness improved from 29.2 % in 2005 to 79.8 % in 2010.

**Table 1:** Completeness: the number of procedures by type of surgical procedure and year and proportion of the procedures in NOIS versus NPR, September - November 2005–2010

	2005	2006	2007	2008	2009	2010	Total
CABG							
NOIS	167	599	680	718	746	612	3522
NPR	1067	1006	1046	928	817	796	5660
*Completeness*	*15.7 %*	*59.5 %*	*65.0 %*	*77.4 %*	*91.3 %*	*76.9 %*	*62.2 %*
CSEC							
NOIS	883	1322	1634	1948	2171	2484	10,442
NPR	2210	2304	2443	2513	2509	2586	14,565
*Completeness*	*40.0 %*	*57.4 %*	*66.9 %*	*77.5 %*	*86.5 %*	*96.1 %*	*71.7 %*
HPRO							
NOIS	903	1052	1338	1853	2522	2565	10,233
NPR	2621	2628	2870	2776	3106	3141	17,142
*Completeness*	*34.5 %*	*40.0 %*	*46.6 %*	*66.8 %*	*81.2 %*	*81.7 %*	*59.7 %*
CHOL							
NOIS	166	234	339	342	409	563	2053
NPR	1356	1308	1394	1362	1285	1275	7980
*Completeness*	*12.2 %*	*17.9 %*	*24.3 %*	*25.1 %*	*31.8 %*	*44.2 %*	*25.7 %*
TOTAL completeness	*29.2 %*	*44.3 %*	*51.5 %*	*64.1 %*	*75.8 %*	*79.8 %*	*57.9 %*

NOIS: Norwegian Surveillance System for Antibiotic Consumption and Healthcare-Associated Infections

NPR: Norwegian Patient Register

**Fig. 1:**
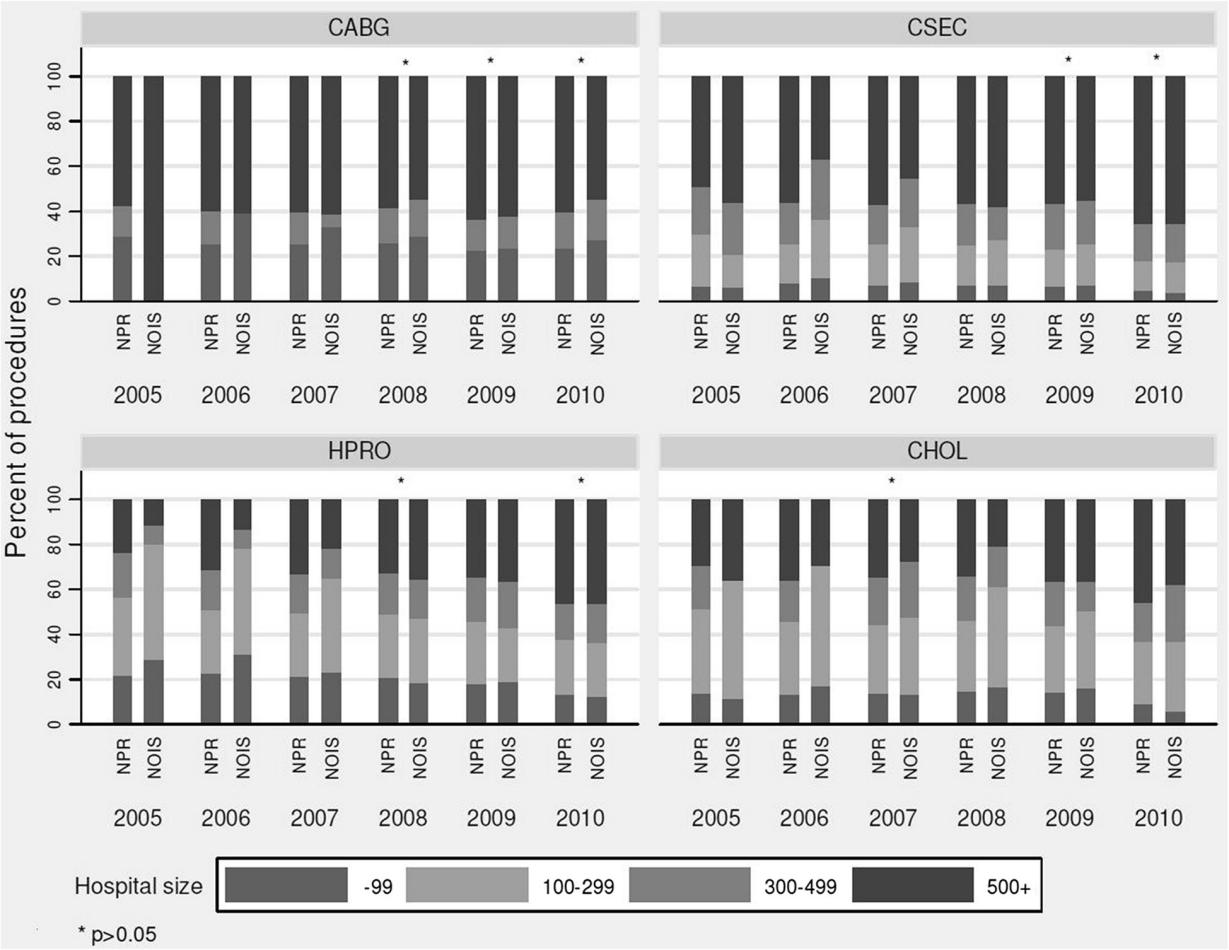
Representativeness: proportion of procedures (in %) by hospital size in NOIS and NPR (2005–2010)

Figure 1 shows the representativeness of NOIS-SSI by comparing the distribution of the procedures in NOIS-SSI with NPR by hospital size for each year. During the first years of operation NOIS-SSI differed significantly from NPR. As more hospitals submitted data during the subsequent years the distributions became more similar and thus more representative for most procedures. There was similar pattern by hospital type (data not shown), and the differences between registers cease to be significant for CABG from 2008 and for CSEC from 2009. For HPRO, only 2009 had no significant differences between the registers. For CHOL the differences are significant for all years by hospital type. By region (data not shown) the differences in distribution between NOIS-SSI and NPR were greater. Only CABG in 2008 and 2009 and CSEC in 2010 had no significant differences. There were no significant differences in distribution by age and sex between NOIS-SSI and NPR (*p* > 0.05). The median age was about 66 for CABG, 31 for CSEC, 73 for HPRO and 49 for CHOL.

Table 2 shows the accuracy of NOIS-SSI compared with NPR by surgical procedure and year, for hospitals and reporting months which were present in both registers. Overall accuracy was 94.8 %, the lowest was 2008 with 90.6 % and the highest was 2010 with 97.5 %. The procedures with the highest overall accuracy were HPRO and CSEC. There were no significant differences in distribution by region, hospital type and size, age or sex for each year and procedure (*p* > 0.05) between NOIS-SSI and NPR.

**Table 2:** Accuracy: the number of procedures by type of surgical procedure and year and proportion of the procedures in NOIS versus NPR for selected hospitals and reporting months, 2005–2010

	2005	2006	2007	2008	2009	2010	Total
CABG^1^							
NOIS	167	402	519	580	503	520	2691
NPR	237	446	589	709	514	554	3049
*Accuracy*	*70.5 %*	*90.1 %*	*88.1 %*	*81.8 %*	*97.9 %*	*93.9 %*	*88.3 %*
CSEC							
NOIS	883	1304	1607	1826	2051	2402	10,073
NPR	904	1346	1660	2014	2065	2431	10,420
*Accuracy*	*97.7 %*	*96.9 %*	*96.8 %*	*90.7 %*	*99.3 %*	*98.8 %*	*96.7 %*
HPRO							
NOIS	903	1052	1338	1853	2151	2335	9632
NPR	943	1087	1451	1959	2194	2363	9997
*Accuracy*	*95.8 %*	*96.8 %*	*92.2 %*	*94.6 %*	*98.0 %*	*98.8 %*	*96.3 %*
CHOL^1^							
NOIS	159	234	339	341	405	524	2002
NPR	194	274	359	395	464	582	2268
*Accuracy*	*82.0 %*	*85.4 %*	*94.4 %*	*86.3 %*	*87.3 %*	*90.0 %*	*88.3 %*
TOTAL accuracy	*92.7 %*	*94.9 %*	*93.7 %*	*90.6 %*	*97.6 %*	*97.5 %*	*94.8 %*

NOIS: Norwegian Surveillance System for Antibiotic Consumption and Healthcare-Associated Infections

NPR: Norwegian Patient Register

^1^Mixed procedures excluded from NOIS for CABG in 2008 and for CHOL in 2007 and 2008

**Fig. 2:**
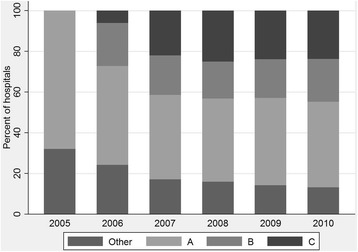
Proportion of hospitals submitting data to NOIS from different electronic systems (**a**, **b** and **c**) and other data sources, 2005–2010

Figure 2 shows the development of ICMs from one major supplier and several manual and in-house systems in 2005, to most data from major ICM suppliers in 2010. All ICMs and other systems in the hospitals perform well, and we only find significant differences between NOIS-SSI and NPR for CSEC in 2008 (*p* = 0.001). System B had the highest overall accuracy (97.5 %). The three commercial systems demonstrate less variability than manual/other systems but the differences were not significant (*p* > 0.05).

## Discussion

The Norwegian Surveillance System for Antibiotic Consumption and Healthcare-Associated Infections (NOIS-SSI) included 79.8 % of the procedures in the administrative data during September-November 2010, up from 29.2 % in 2005. NOIS-SSI was not representative with regard to hospital size and type during the earliest years, but became representative with time for some procedures. NOIS-SSI was representative with regard to age and sex for all years and procedures. The accuracy was 97.5 % in 2010, an increase from 92.7 % in 2005 and there were no differences in the distribution by any explanatory variables, except by type of infection control module (ICM) for CSEC in 2008.

Comparing denominator data between two registers gives an indication of the quality of the data in both registers. It also reflects the quality of the data extraction at the individual hospital. Denominator data are important in order to reliably describe infection occurrence on a national level, in hospital benchmarking, and inter-country comparisons. Regardless of how diligent numerator (infection) case finding is, incidence proportions only make sense if the denominator data are correctly derived, giving an unbiased sample [13]. In a recent review, Goto [14] investigated the accuracy of administrative coding, but none of the included SSI-related studies reported on the quality of denominator data. McCoubrey [15] found that 91 % of eligible procedures were included in the Scottish surveillance data. Haley [16] found 98 % matches between administrative- and surveillance data. Most validation studies report only on the numerator in terms of infection as outcome. A number of studies have investigated the completeness of other Norwegian health registers compared with NPR. Among these, 0.4 % more CSECs were found in the Medical Birth Register of Norway [17], the Norwegian Vascular Register found a completeness of 84 % for abdominal aortic aneurism repair [18], and the Norwegian Arthroplasty Register found 97 % completeness of primary HPRO compared with NPR [19]. These studies are important because in addition to ascertaining the quality of the individual registers, validate the quality of NPR. Although it has improved, NOIS-SSI still only received 78.8 % of the procedures performed during September-November 2010.

Because NOIS-SSI only collected data during September-November during the study period, it was dependent on those 3 months being representative. The explanatory variables which reflect hospital participation (region, hospital type and size), show that NOIS-SSI was generally not representative for most procedures until the last years. There are several possible explanations for this.

During the first years, many hospitals were granted exemption from submitting data in order to facilitate the establishment of ICMs. The ICMs were generally purchased or developed for whole trusts or regions, which led to several regions submitting little or no data during the first years. Most hospitals and regions had installed ICMs by 2007, but some were not functioning optimally. This led to some hospitals and trusts being exempted also in the later years, and NOIS-SSI not being representative by region.

During 2005–2009 NOIS-SSI only required data from one procedure, the one with the highest priority. This means that hospitals were only required to submit data from the highest prioritized procedure which they performed. All hospitals which performed CABG procedures were required to submit data, but exemptions were granted to some regions and hospitals the first years. In addition, some hospitals did not submit data in later years despite it being required. If a hospital reported on CABG, it did not have to submit any other procedures. In principle this meant that none of the tertiary hospitals, which almost all performed CABG, were required to submit CSEC data causing poor representativeness by type of hospital for CSEC. This also affected representativeness by hospital size, because the tertiary hospitals are generally the largest. From 2010 a minimum of 2 procedures were required and this improved the representativeness for CSEC by hospital size and type. However, CSEC representativeness was already good in 2009, probably attributable to “enthusiastic volunteers”. For HPRO, representativeness by hospital size started improving in 2008. Some of the hospitals which perform HPRO are specialized orthopedic hospitals, and these have submitted data consistently over the years. Many other hospitals have submitted HPRO data voluntarily, and this may explain why representativeness started improving before the implementation of minimum 2 procedures in 2010. For CHOL representativeness was generally poor, which is to be expected as this procedure had the lowest surveillance priority. For age and sex NOIS-SSI was representative, meaning that there were no differences between NOIS-SSI and NPR in the patient population for these variables.

In a review of four surveillance systems Haustein et al. [20] recommended mandatory reporting in order to assure that data are not biased. They found that none of the voluntary systems they investigated ever surpassed 50 % participation, and that representativeness improved when reporting was made mandatory. NOIS-SSI was mandatory since inception, but a flexible implementation policy (granting exemptions) caused it not to be representative on a national level and caused participant population to change over time. The additional complication of hospitals changing from individual hospital to trust level reporting produces data which is less useful for stratification and risk purposes. This is demonstrated in Fig. 1, where a greater proportion of large hospitals are evident during the latter years. For example, 2 small primary hospitals and 1 large tertiary hospital reported individually until 2008 and from 2009 they reported as one large trust on the tertiary level.

The importance of representative surveillance data depends on how data are to be used. For evaluating risk factors and implementing preventive measures in the individual hospitals, NOIS-SSI seems to provide useful data. For hospital benchmarking and/or public reporting NOIS-SSI was not good enough, because when hospitals are not required to submit all procedures, full representativeness for such variables as hospital size and type may not be achieved.

We found the agreement between the two registers to be good, which means that when the hospitals did submit data to NOIS-SSI they appeared to be accurate. We only observed a significant difference (*p* = 0.001) between the registers by ICM for CSEC in 2008, which was mainly due to technical issues in two hospitals with the same ICM-supplier resulting in incomplete data extraction. Another reason for somewhat lower accuracy in some procedures and years was that the NOIS-SSI protocol was modified with regard to mixed procedures. The exclusion of the mixed CABG procedures in 2008 gave a dip in the accuracy of NOIS-SSI (not significant). For CHOL, exclusion of mixed procedures did not appear to influence accuracy, which is reasonable because over 90 % of CHOLs were laparoscopic procedures [21] and generally not mixed (Table 2).

Automated data collection is becoming a very important tool in surveillance of HAI. It reduces the workload on hospital staff and, hopefully, human errors [22–31]. In NPR all data are collected electronically from the hospitals’ EHR and in NOIS-SSI most explanatory and background variables are collected electronically from the EHR, so we could expect denominator data to be identical. However, data extraction programs may not be identical in all systems, and the syntax may differ in the way data are extracted and interpreted. In addition, NOIS-SSI data are manually checked by infection control practitioners who may manually correct the data. As demonstrated by the lower accuracy in CSEC for 2008, one cannot be certain that denominator data are correct even if they are extracted directly from hospital computer systems. Computer systems are not infallible, and it is necessary to routinely check if data are being harvested correctly. We observe some variability between the ICMs and other systems and it appears that the accuracy overall for the ICMs was more consistent than the manual/other systems, but none of the differences were significant.

The development of ICMs is complex and would have been more difficult without a flexible implementation strategy. As shown in Fig. 2, the hospitals quite quickly purchased or developed ICMs. We found the flexible implementation to be a double-edged sword. On one side the flexibility made good cooperation with hospitals and ICM suppliers possible and has led to quality ICMs which give good accuracy. On the other side this flexibility contributed to less representative data. Although NOIS-SSI is mandatory, the flexible implementation introduced selection bias giving poor representativeness for variables that reflect hospital participation.

NOIS-SSI improved over the first six years, but data were still not fully complete and representative in 2010. The accuracy of NOIS-SSI was good, because the hospitals which submitted data have had consistently good denominator quality throughout the years, with a few exceptions. We also saw an indication that automated data harvesting gave slightly better denominator data quality. It is, however, difficult to assess true completeness, representativeness and accuracy without having access to linked data [32]. Being able to compare surveillance data with administrative data on a regular basis, in order to give hospitals feedback on data quality, could be a useful tool in improving quality and instilling trust in the surveillance system performance. Some have argued that administrative systems can provide more economical, standardized and unbiased outcome data than traditional surveillance systems if used correctly [33–35].

The data in this study are not linked and are compared on an aggregated level. We cannot be certain that NOIS-SSI is a subset of NPR, as both registers may contain unique records. Some variables were coded manually by the authors, and may contain unintentional errors. Birth month and date for the NPR data were generated by a pseudo-random function and does not reflect different annual birth rate patterns. For calculation of accuracy some hospitals and months were excluded from analysis, and this may give an incorrect impression of the quality of NOIS-SSI.

## Conclusions

NOIS-SSI had a completeness of 79.8 % of the procedures in the administrative data (NPR). The NOIS-SSI denominator data were not representative by hospital size and type during the first years of surveillance system operation, but became representative for some procedures with time. NOIS-SSI was generally not representative by region. This means that data from this period should not be used for hospital benchmarking and/or public reporting. NOIS-SSI was representative by age and sex for all procedures. For the purpose of evaluating risk factors and implementing prevention and precautionary measures in the individual hospitals, representativeness seems sufficient. Denominator data agreement between NOIS-SSI and NPR of almost 95 % indicates that the accuracy of submitted data of was good. A flexible and incremental implementation strategy has encouraged development of computer-based surveillance systems in hospitals which gives good accuracy, but has adversely affected the representativeness of the data during the first years of system operation.

## Abbreviations

CABG: Coronary artery bypass graft
CHOL: Cholecystectomy
CSEC: Cesarean section
ECDC: European Centre for Disease Prevention and Control
EHR: Electronic health record
HPRO: Hip arthroplasty
ICM: Infection control module
NCSP: Nordic Medico-Statistical Committee’s Classification of Surgical Procedures
NIPH: Norwegian Institute of Public Health
NOIS: Norwegian Surveillance System for Antibiotic Consumption and Healthcare-Associated Infections
NPR: Norwegian Patient Register
SSI: Surgical site infection
